# Supplementary material for: Identification of multiple Acinetobacter baumannii protein antigens as targets for potential immunotherapies using a novel protein microarray screening approach
Source: PLoS Pathog. 2026 Feb 12;22(2):e1013958. doi: 10.1371/journal.ppat.1013958 (PMC12919932; doi:10.1371/journal.ppat.1013958)
Supplement: S1 Fig — (A and B) Mice (n = 6) were passively immunised by intraperitoneal (IP) inoculation of 50 μg rabbit polyclonal IgG or PBS 4 h before IP inoculation with 3–6 x 10^6^ CFU/mouse of strain AB3879 suspended in PBS with 5% porcine mucin. Dot plots represent bacterial CFU in the indicated target organs from individual mice (bars = means, error bars = SDs) 20–24 h after IP infection. Data were analysed using Kruskal-Wallis one-way analysis of variance compared to PBS controls (*p < 0.05, **p < 0.01, ***p < 0.001, ****p < 0.0001, ns; not significant). (C and D) Mean (SDs) C3b/iC3b deposition index (% positive x median fluorescent intensity of bacteria staining positive for C3b/iC3b) after incubation in normal human serum (NHS only), or NHS plus antibody (as indicated), or the secondary antibody alone (2° Only). (DOCX) [file ppat.1013958.s001.docx]

**D**

**B**

**A**

**C**

**S1 Fig. Effects of less protective rabbit IgG to selected antigens. (A and B)** Mice (n=6) were passively immunised by intraperitoneal (IP) inoculation of 50 μg rabbit polyclonal IgG or PBS 4 h before IP inoculation with 3-6 x 10^6^ CFU/mouse of strain AB3879 suspended in PBS with 5% porcine mucin. Dot plots represent bacterial CFU in the indicated target organs from individual mice (bars = means, error bars = SDs) 20-24 h after IP infection. Data were analysed using Kruskal-Wallis one-way analysis of variance compared to PBS controls (**p* < 0.05, ***p* < 0.01, ****p* < 0.001, *****p* < 0.0001, ns; not significant). **(C and D)** Mean (SDs) C3b/iC3b deposition index (% positive x median fluorescent intensity of bacteria staining positive for C3b/iC3b) after incubation in normal human serum (NHS only), or NHS plus antibody (as indicated), or the secondary antibody alone (2° Only).
