# Supplementary material for: Identification of multiple Acinetobacter baumannii protein antigens as targets for potential immunotherapies using a novel protein microarray screening approach
Source: PLoS Pathog. 2026 Feb 12;22(2):e1013958. doi: 10.1371/journal.ppat.1013958 (PMC12919932; doi:10.1371/journal.ppat.1013958)
Supplement: S2 Fig — Mice (n = 6) were injected intraperitoneally (IP) with PBS, α-Ly6G, or an isotype control antibody 24 h prior to passive immunization by IP inoculation of 50 µg of rabbit polyclonal IgG to Ag7, or PBS. 4 h post-immunization mice were inoculated IP with 3–6 x 10^6^ CFU/mouse of strain AB3879 suspended in PBS with 5% porcine mucin. Dot plots represent bacterial CFU in the indicated target organs from individual mice (bars = means, error bars = SDs) 20–24 h after IP infection. Data were analysed using Kruskal-Wallis one-way analysis of variance compared to no treatment controls (*p < 0.05, **p < 0.01, ***p < 0.001, ****p < 0.0001, ns; not significant). (DOCX) [file ppat.1013958.s002.docx]

α-Ag7 - + + + - + + +

α-Ly6G - - + - - - + -

Isotype Ctrl - - - + - - - +

**S2 Fig. Neutrophil depletion prevents passive protection by rabbit antibodies to selected antigens.** Mice (n=6) were injected intraperitoneally (IP) with PBS, α-Ly6G, or an isotype control antibody 24 h prior to passive immunization by IP inoculation of 50 µg of rabbit polyclonal IgG to Ag7, or PBS. 4 h post-immunization mice were inoculated IP with 3-6 x 10^6^ CFU/mouse of strain AB3879 suspended in PBS with 5% porcine mucin. Dot plots represent bacterial CFU in the indicated target organs from individual mice (bars = means, error bars = SDs) 20-24 h after IP infection. Data were analysed using Kruskal-Wallis one-way analysis of variance compared to no treatment controls (**p* < 0.05, ***p* < 0.01, ****p* < 0.001, *****p* < 0.0001, ns; not significant).
