# Supplementary material for: Identification of multiple Acinetobacter baumannii protein antigens as targets for potential immunotherapies using a novel protein microarray screening approach
Source: PLoS Pathog. 2026 Feb 12;22(2):e1013958. doi: 10.1371/journal.ppat.1013958 (PMC12919932; doi:10.1371/journal.ppat.1013958)
Supplement: S3 Fig — Growth of A. baumannii clinical isolates over 24 h at 37°C in LB media (A) or LB plus 4 µg/ml colistin (B) (means, error bars = SDs, n = 3). MIC ≥ 4 µg/ml = resistant to colistin in accordance with CLSI MIC Breakpoints 2023. KL = K locus capsule type. (DOCX) [file ppat.1013958.s003.docx]

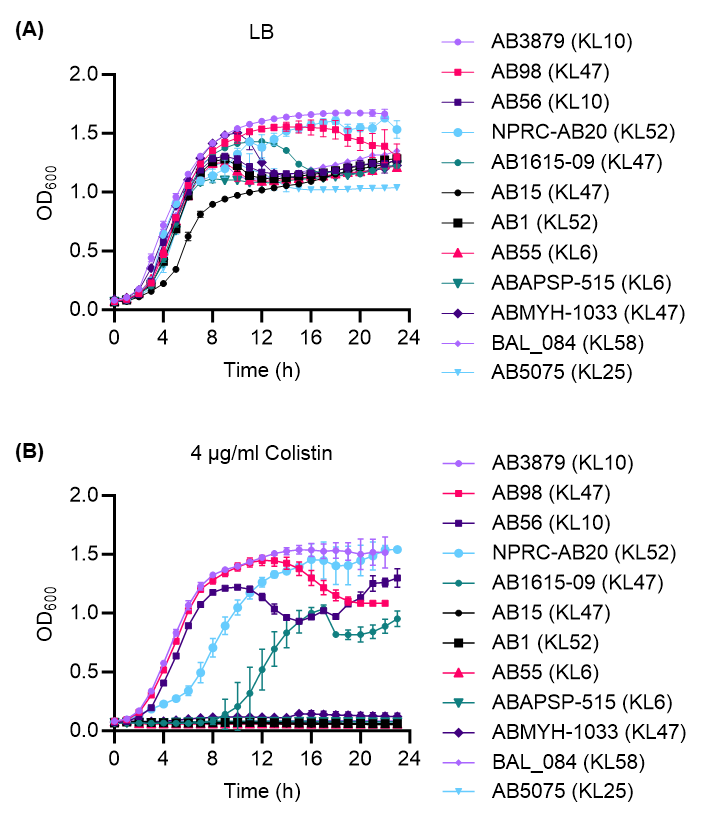


**S3 Fig. Colistin resistance data for clinical *A. baumannii* isolates.** Growth of *A. baumannii* clinical isolates over 24 h at 37°C in LB media **(A)** or LB plus 4 µg/ml colistin **(B)** (means, error bars = SDs, n=3). MIC ≥ 4 µg/ml = resistant to colistin in accordance with CLSI MIC Breakpoints 2023. KL = K locus capsule type.
