# Supplementary material for: Identification of multiple Acinetobacter baumannii protein antigens as targets for potential immunotherapies using a novel protein microarray screening approach
Source: PLoS Pathog. 2026 Feb 12;22(2):e1013958. doi: 10.1371/journal.ppat.1013958 (PMC12919932; doi:10.1371/journal.ppat.1013958)
Supplement: S2 Table — (DOCX) [file ppat.1013958.s005.docx]

**S2 Table: *Acinetobacter baumannii* clinical isolates and their antibiotic**

**sensitivities (R = resistant).**

|  |  |  |  | **Antibiotic Resistance Profile^δ^** | | | | | | | | | | | |
| --- | --- | --- | --- | --- | --- | --- | --- | --- | --- | --- | --- | --- | --- | --- | --- |
| **Strain^α^** | **KL^β^** | **ST ^γ^** | **Source** | **β-lactams** | **Carbapenems** | **Colistin** | **Aminoglycosides** | **Macrolides** | **Tetracyclines** | **Rifampicin** | **Trimethoprim** | **Sulphonamides** | **Chloramphenicol** | **Fluoroquinolones** | **Cephalosporins** |
| AB15^1^ | KL47 | ST2 | Sputum | R | R | S | R | R | R | ND | R | R | ND | R | R |
| AB98^1^ | KL47 | ST215 | Unknown | R | R | R | R | R | R | ND | ND | R | R | ND | ND |
| AB1615-09^2^ | KL47 | ST2 | Sputum | R | R | S | R | ND | R | ND | ND | ND | ND | ND | ND |
| AB56^1^ | KL10 | ST2 | Unknown | R | R | R | R | R | R | ND | ND | R | ND | ND | ND |
| AB3879^2^ | KL10 | ST2 | Sputum | R | R | R | R | R | R | R | R | R | R | R | R |
| NPRC-AB20^2^ | KL52 | ST2 | Unknown | R | R | R | R | R | R | ND | R | R | R | R | R |
| AB1^1^ | KL52 | ST2 | Pus | R | R | S | R | R | R | ND | R | R | ND | R | R |
| AB55^1^ | KL6 | ST2 | Unknown | R | R | S | R | R | R | ND | ND | R | ND | ND | ND |
| ABAPSP-515^3^ | KL6 | ST164 | Sputum | R | R | S | ND | ND | R | ND | ND | ND | ND | ND | ND |
| ABMYH-1033^3^ | KL47 | ST2 | Blood | R | R | S | R | ND | R | ND | ND | R | ND | ND | ND |
| BAL_084^4^ | KL58 | ST2 | Sputum | R | R | S | R | ND | ND | ND | ND | ND | ND | R | R |
| AB5075^5^ | KL25 | ST1 | Wound | R | R | S | R | R | ND | ND | R | ND | R | R | ND |
| AB5075*^wza^* ^5^ | N/A | ST1 | N/A | R | R | S | R | R | ND | ND | R | ND | R | R | ND |
| AB5075*^ag1^* ^6^ | KL25 | KL25 | N/A | ND | ND | ND | ND | ND | ND | ND | ND | ND | ND | ND | ND |
| AB5075*^ag5^* ^5^ | KL25 | KL25 | N/A | ND | ND | ND | ND | ND | ND | ND | ND | ND | ND | ND | ND |
| AB5075*^ag7^* ^5^ | KL25 | KL25 | N/A | ND | ND | ND | ND | ND | ND | ND | ND | ND | ND | ND | ND |
| *E. coli* MFDpir^6^ | N/A | N/A | N/A | ND | ND | ND | ND | ND | ND | ND | ND | ND | ND | ND | ND |

^α^Origin: ^1^Siriraj Hospital, Bangkok, Thailand, ^2^Songklanagarind Hospital, Songkhla, Thailand, ^3^Thammasat University Hospital, Pathum Thani, Thailand, ^4^Hospital for Tropical Diseases, Ho Chi Minh, Vietnam, ^5^Manoil Laboratory, University of Washington, USA, ^6^this study (see Materials and Methods).

^β^K Locus (KL) Capsule Types.

^γ^Pasteur Multilocus Sequence Type (MLST) sequence type (ST) number.

^δ^R = antimicrobial resistance inferred from genotypic and/or phenotypic data, based on the presence of antimicrobial resistant genes (Aac3-Iia, Aac3-I, AacAad, AadA, AadB, Aph3-Ia, Aph4-Ia, AphA6, ArmA, RmtB, StrA, StrB, BlaA1, BlaA2, CARB, Imp-1, Mbl, NDM-1, OXA-237, OXA-23, OXA-24, OXA-48, OXA-51, OXA-58, OXA-7, Per-1, OKP-A-1, TEM-1D, VEB-1, Zn-dependent hydrolase, beta-lactamase class C, CatA1, CatBx, CmlA, FloR, Dfr16, DfrA10, DfrA27, DfrA5, MphA, MphE, MsrE, Sul2, Sul1, TetA, TetB, TetR, TetY, Tet-39, Arr) (20), or confirmed from MIC data (**S3 Fig** and (33,35,38). S = sensitive, ND = not determined.
