# Supplementary material for: Identification of multiple Acinetobacter baumannii protein antigens as targets for potential immunotherapies using a novel protein microarray screening approach
Source: PLoS Pathog. 2026 Feb 12;22(2):e1013958. doi: 10.1371/journal.ppat.1013958 (PMC12919932; doi:10.1371/journal.ppat.1013958)
Supplement: Attachment — Submitted filename: PPATHOGENS-D-25 ResponseToReviewers.docx [file ppat.1013958.s008.docx]

**PPATHOGENS-D-25-01927**

**Identification of multiple *Acinetobacter baumannii* protein antigens as targets for potential immunotherapies using a novel protein microarray screening approach**

**Palethorpe *et al.***

**Reply to reviewers’ comments**

Thank you for the reviews of the above manuscript. We have addressed each of the editor’s and reviewer’s comments as outlined below. This has involved repeating Western immunoblots, ELISAs and additional animal model experiments and inclusion of completely new data on active vaccination, along with multiple text changes (indicated below by line numbers corresponding to the “Marked Up” version of the Main Text). We feel these changes have considerably improved the manuscript and hope it is now acceptable for publication in PLOS Pathogens.

***Editors overview:*** *we invite you to submit a substantially revised version of the manuscript that addresses all of the points raised by the three reviewers. In particular, you need:*

*1. additional discussion of the biological functions of the antigens:*

We have added text to the discussion providing further description of the putative biological functions of our target antigens (**lines 384-400**).

*2. clarification of experimental details,*

Additional text on experimental details has been added at several points in the manuscript as outlined below in the reply to reviewers’ comments.

*3. controls that include whether LPS is present in the vaccine preparations,*

We have measured LPS in our protein preparations and have edited the text in the manuscript (**lines 547-48**); how the results may affect the data is discussed below in reply to comments from Reviewers.

*4. and publicly available RNA-seq data.*

We apologise for this oversight - the RNAseq data had previously been uploaded to a public repository and is referenced in a previous paper; the links have now also been added to the methods of this manuscript (**lines 520-24**).

*5. Insert an Author Summary.*

This is now added just after the Abstract.

**Responses to Reviewers' Comments:**

Major comments (paraphrased for brevity when necessary):

*1. Was LPS removed from the protein preparations? If not, LPS contamination can affect immune responses in vivo.*

We have now tested the purified proteins using the Pierce Chromogenic Endotoxin Quant Kit, which did detect LPS contamination (**lines 547-48**). LPS from a different species is unlikely to induce protective antibody against *A. baumannii*, but to formally demonstrate this we have now included additional data showing that passive immunisation with rabbit polyclonal antibody to two additional protein antigens failed to protect against AB3879 sepsis despite these protein preparations also containing LPS (**Supplementary Figure 1 panels A and B, text lines 284-89 and 332-35**).

*2. Were rabbits immunized with the protein antigens alone, or with an adjuvant, to generate antibodies?*

Polyclonal IgG to the selected antigens was obtained from ThermoFisher, who use rabbit immunisations with the target protein and Complete and Incomplete Freund’s Adjuvant as the adjuvant. This has been added to the text at **lines 544-46.**

*3. The number of animals per group, the sex of the animals, and whether the experiments were repeated, should be included in the legends of each figure.*

Mouse experiments with significant results were repeated at least twice (**stated in lines 612-14**). Exact n numbers have been added to figure legends where missing. Our initial pilot experiments included both male and female mice, however, since no sex differences were observed, subsequent experiments were conducted using only female mice to minimize animal use, in accordance with the 3R’s (replacement, reduction, refinement). The sex of the animals is indicated in the materials and methods (**lines 571-72, 581, and 589**).

*4. Figure 2A will be easier to read if purified antigens and bacterial lysates were run on the same gel next to each other …. And each lane labelled with the name of the lysate,*

As suggested, we have repeated these Western blots with protein and three *A. baumannii* strain lysates side by side and including transposon mutants for Ag1, 5 and 7 (Ag11 is essential) (**Figure** **3**). The text has been adjusted accordingly (**lines 200-10**).

*5. ELISA assays use no sera as the negative control. Inclusion of serum from a cytoplasmic protein, or unrelated antigen would be a better control.*

We agree; however as the polyclonal antibodies were obtained commercially we do not have any antibody to an unrelated protein, and antibody to cytoplasmic proteins also gives positive whole cell ELISA data as internal proteins are exposed when the bacteria are fixed to the plates. In the absence of these controls, we repeated the ELISAs with purified polyclonal IgG derived from the rabbit antisera which should give a cleaner result than using the rabbit anti-sera. These data are presented in **Figure 4** and gave similar results to the rabbit sera ELISAs. Importantly, the immunoblots in **Figure 3** showed that polyclonal IgG failed to recognise mutant strains missing the target antigen for antigens 5 and 7 (antigen 11 is essential so no mutant is available). This demonstrates that the binding of these polyclonal IgG in the ELISAs is likely to represent binding to their specific antigen rather than non-specific binding. For antigen 1 some non-specific bands remained, and these could conceivably have affected the results of the ELISAs; text on these issues has been added in **lines 208-10.**

*6. Treatment of a group of mice with isotype control antibody along with anti-Ly6G is an important control for the depletion experiments.*

We agree, and have repeated the neutrophil depletion experiment using an isotype control and these data has been added to **Supplementary Fig. 2** and the relevant text adjusted in **lines 325 and 592**.

*7. It is important to test whether active immunization with purified proteins generates protective immune responses in mice.*

We agree, and have now added data on the utility of our selected antigens in active vaccination experiments (**new Figure 7**). The results show that vaccination with our selected antigens induced antigen-specific IgG responses, and that could (for some antigens) protect against *A. baumannii* sepsis. Text on these findings and the methods have been added in **lines 304-14, 363-65, 412-13, 581-88**.

*8. The histograms in Figure 5 should be labeled to improve readability of the figure.*

We have relabelled these histograms and hope the changes make this figure more easily interpreted (**Figure 5**).

**Part II – Major Issues: Key Experiments Required for Acceptance**

*Reviewer #1. A detailed analysis and interpretation of the broth versus ex vivo serum RNA-Seq experiments would be extremely useful to the field*

*And*

*It would be useful to describe what the three candidates are in more detail and their potential roles in the cell, fitness or pathogenicity*

We agree these analyses are important, and have a second manuscript in preparation that describes in detail the RNAseq data and further characterisation of the antigens of interest. These are extensive datasets, and we feel are more appropriately presented as a second paper rather than incorporated into this manuscript which already includes a considerable amount of data. We agree that further description of the potential role of our selected antigens should be added to this manuscript and have added text on this in **lines 384-400**.

*Reviewer #2: None*

*Reviewer #3:*

*Testing LPS content in the purified proteins.
Isotype control antibody treatment of mice as a control for neutrophil depletion.
Active immunization with the recombinant proteins to demonstrate protection.*

As described above we have now added new experimental data to address these issues.

**Part III – Minor Issues: Editorial and Data Presentation Modifications**

*Reviewer #1:*

*• Throughout the study different strains are used for different assays, more rationale is needed each time a strain is dropped or included in each assay for example Line 217 why select these two strains here? And why for example were AB5075 and the corresponding wza mutant not included here?*

We have added more text to make the rationale why various strains were used at different points in the manuscript (**lines 201-3, 213-15, 231, and 245-47**). In general, AB5075 and its unencapsulated derivative *wza* were used to show the effect of the capsule on immune assays, whereas comparison of immune recognition data between strains concentrated on clinical isolates from Thailand as these will be the target for any future antibody therapy.

*• Line 200 Clarify what KL type AB3879 is here …... needs to be clearly identified in each of the relevant figure legends.*

The text and figure legends have been adjusted accordingly.

*• Line 208 rationale should be given for the differences particularly when independent of KL type. Needs more interpretation in the discussion also.*

*And*

*• Line 232 reasons for the lack of alignment should be briefly mentioned here and expanded upon in more detail in the discussion.*

There is no clear answer to why the results of ELISA, IgG opsonisation, complement binding, and MAC formation are not all directly correlated; this is likely to be due to differences in antigen expression levels, surface accessibility between strains, porosity of the capsule, and capsule effects on IgG and complement opsonisation and MAC activation. This has now been mentioned in the results (**lines 264-67**), and the discussion has been expanded to describe this further (**lines 441-49**).

*• Line 240 more detail needed here. Why was this strain selected when mucin needed to support colonisation? Citation(s) needed here.*

Animal models of *A. baumannii* infection are not straight forward as many clinically relevant *A. baumannii* isolates have low virulence in immunocompetent mice. Injection of mucin is an established method for overcoming this; the text has been altered and references inserted as suggested to describe this more clearly **(lines 269-72)**. None of the clinical isolates we have tested are able to cause sustained systemic infection without addition of mucin. Strain AB3879 was used as it reliably develops sepsis when injected with mucin, and was the strain used in our previous publications (**references 33 and 35 from the paper**).

*• Line 310 previously described as ex vivo serum?*

This has been amended (**now line 370**).

*• The RNA-Seq data does not appear to have been deposited in an open access data for reviewer analysis.*

We apologise for this oversight - this was previously published in our earlier paper, and we have now repeated this in **lines 520-24.**

*• Include a statement that the Thai strains are available to the wider research community upon request or are deposited in a centralized collection*.

We have now added to **line 497-98** that the clinical strains described in this manuscript are available on request to the corresponding author.

*Reviewer #2:*

*• Explain why they stated dimers were formed for Ag5*

Denaturing SDS gels were used for the immunoblots (now stated in the text **lines 200 and 548-49**). We have repeated the immunoblots and the dimer bands are much less obvious than previously, and hence we have removed the text discussing this from the manuscript.

*• Comment on the essentiality of the proteins that were tested.*

BamA (Ag11) is known to be essential, and we have added this to the text (**lines 195 and 208**). The data on the phenotypes of mutants affecting the other selected antigens will be described in a second paper; text on their potential roles is inserted in **lines 384-400**.

*• The authors could also expand the discussion to include a more detailed comparison between the human and mouse serum microarray results.*

This is an interesting and important point. However, although we have extensive data on screening human sera from infected subjects against the protein microarray, these data are beyond the scope of this paper as they largely pertain to the diagnostic utility of human serological responses rather than to antigen discovery and validation. Hence, we are not able to compare the serological data from mice and humans in this manuscript.

*• Could the identified candidates be used in combinatorial immunotherapy?*

Yes, we actually aim to do this, and this was partially addressed by the original **Figure 6** showing data for combining passive administration of Ag1 and Ag11, and is discussed in **lines 431-33**.

*• How might prophylactic treatment be administered prior to confirmed infection?*

*And*

*• What is the intended target population for such therapies?*

Prophylactic antibody could be given to subjects for which clinical data show are at a high risk of infection, for example patients ventilated on intensive care in Southeast Asia hospitals as these patients have a high incidence of *A. baumannii* pneumonia. Therapeutic antibody could also be given to patients with confirmed *A. baumannii* infection in combination with antibiotics to try and improve outcomes. We have added additional text on this in **lines 345-49**.
