# Supplementary material for: Identification of multiple Acinetobacter baumannii protein antigens as targets for potential immunotherapies using a novel protein microarray screening approach
Source: PLoS Pathog. 2026 Feb 12;22(2):e1013958. doi: 10.1371/journal.ppat.1013958 (PMC12919932; doi:10.1371/journal.ppat.1013958)
Supplement: Attachment — Submitted filename: PPATHOGENS-D-25-01927 ResponseToReviewers.docx [file ppat.1013958.s009.docx]

**PPATHOGENS-D-25-01927**

**Identification of multiple *Acinetobacter baumannii* protein antigens as targets for potential immunotherapies using a novel protein microarray screening approach**

**Palethorpe *et al.***

**Reply to reviewers’ comments**

Thank you for the minor revisions suggested for the above manuscript. We have addressed each of the comments as outlined below (indicated by line numbers corresponding to the “Marked Up” version of the Main Text). We hope it is now acceptable for publication in PLOS Pathogens.

1. *[ Supplementary Table 2] : appears to have been adapted from a previously published figure. Please provide written permission from the copyright holder to publish this under our CC-BY 4.0 license, or remove the figure / replace the image. Please note we do not recommend using standard request forms available on Publishers' websites, as they grant single use rather than republication under an open access license.*

Supplementary table 2 was made new for this paper as it includes all the antibiotic resistance data for these strains including new experimental data that has not been represented before. It is true that most of the strains have been listed before in one of our previous papers, however that must be the case for a lot of manuscripts, and in fact we have added strains as well. Our phrase ‘adapted from reference 33’ is actually inaccurate and has therefore been removed.

2. *We do not publish any copyright or trademark symbols that usually accompany proprietary names, eg ©,  ®, or TM  (e.g. next to drug or reagent names). Therefore please remove all instances of trademark/copyright symbols throughout the text, including:  Bexsero®. on line no 778.*

This has now been removed.

3. *Thank you for stating "All mouse experiments were performed at UCL following national 546 guidelines and were approved by the UCL Biological Services Ethical Committee and the UK Home Office (project number PP3031297)."
   Please state the full name of UCL*

This has been corrected (now lines 549 - 50).

4. *Please ensure that the funders and grant numbers match between the Financial Disclosure field and the Funding Information tab in your submission form. Note that the funders must be provided in the same order in both places as well.
    Currently, " The BactiVac network grant BVNCP6-03" is missing from the Funding Information and also the order is different in both the places*

This has been corrected.

5. *Report the amount of LPS present in the methods section as requested by Reviewer 3*

This has now been added (lines 530 - 532).

6. *Correct the typo in the Author Summary, should be "One important AMR bacterium"*

This has been corrected (line 62).

7. *Line 285 (marked): "polyclonal IgG antibodies"?*

This has been corrected (now line 283).

8. *Line 515 (marked): Revise to more clearly indicate that the RNAseq datasets were previously published, e.g. "Transcriptome analyses by RNAseq were previously described"*

This has been corrected (now line 499).
